# MODIS Based Estimation of Forest Aboveground Biomass in China

**DOI:** 10.1371/journal.pone.0130143

**Published:** 2015-06-26

**Authors:** Guodong Yin, Yuan Zhang, Yan Sun, Tao Wang, Zhenzhong Zeng, Shilong Piao

**Affiliations:** 1 College of Urban and Environmental Sciences, Peking University, Beijing, China; 2 Institute of Tibetan Plateau Research, Chinese Academy of Sciences, Beijing, China; Chinese Academy of Forestry, CHINA

## Abstract

Accurate estimation of forest biomass C stock is essential to understand carbon cycles. However, current estimates of Chinese forest biomass are mostly based on inventory-based timber volumes and empirical conversion factors at the provincial scale, which could introduce large uncertainties in forest biomass estimation. Here we provide a data-driven estimate of Chinese forest aboveground biomass from 2001 to 2013 at a spatial resolution of 1 km by integrating a recently reviewed plot-level ground-measured forest aboveground biomass database with geospatial information from 1-km Moderate-Resolution Imaging Spectroradiometer (MODIS) dataset in a machine learning algorithm (the model tree ensemble, MTE). We show that Chinese forest aboveground biomass is 8.56 Pg C, which is mainly contributed by evergreen needle-leaf forests and deciduous broadleaf forests. The mean forest aboveground biomass density is 56.1 Mg C ha^−1^, with high values observed in temperate humid regions. The responses of forest aboveground biomass density to mean annual temperature are closely tied to water conditions; that is, negative responses dominate regions with mean annual precipitation less than 1300 mm y^−1^ and positive responses prevail in regions with mean annual precipitation higher than 2800 mm y^−1^. During the 2000s, the forests in China sequestered C by 61.9 Tg C y^−1^, and this C sink is mainly distributed in north China and may be attributed to warming climate, rising CO_2_ concentration, N deposition, and growth of young forests.

## Introduction

Forests contain about 80% of global terrestrial aboveground biomass (AGB), and play a key role in the global carbon cycle [1,2]. It has been estimated that forest ecosystems have sequestered annually 1.1 Pg of the carbon over the last two decades [3], which is nearly about 16% of the carbon released by fossil fuel CO_2_ emissions during the same period [4]. However, there is evidently considerable uncertainty about the magnitude of the forest carbon sink, and even larger uncertainty about its location. Much of this uncertainty is attributed by the incomplete information regarding the spatial distribution of carbon stored in biomass [5,6]. Therefore, it is critically important to improve knowledge of the density and spatial distribution of forest biomass for supporting future climate mitigation actions [2].

Terrestrial ecosystems in China have been absorbing 28–37% of fossil carbon emissions during the 1980s and 1990s, with much of this uptake occurring via carbon accumulation in forest biomass [7]. Previous estimation of forest biomass and its change in China is predominantly based upon periodical national-level forest resource inventories [8–10]. However, the spatial distribution of aboveground biomass is not well characterized since these inventories only provide the information on different forest types at the provincial level [9]. In addition, inventory-based approaches simply convert forest carbon biomass from inventory variables, such as timber volumes, by applying an empirical factor (e.g., biomass expansion factor, BEF) [9], which introduces large uncertainties in the estimation of forest biomass. For example, due to use of different volume-biomass relationship (or different value of BEF), the estimates of China forest biomass by Pan et al. [3] is smaller by 15–27% than previous estimates by Fang, et al. [11], although both studies used the same inventory data. Pan et al. [3] also suggested that separating age groups with the volume–biomass method could cause 89% difference in carbon sequestration rate in China.

Remote sensing has been extensively used as a basis for mapping aboveground forest biomass [12–15]. There is ample evidence that demonstrates the general sensitivity of spectral reflectance particularly in the shortwave infrared bands to vegetation structure, which is correlated with aboveground forest biomass [13–16]. For example, Piao, et al. [17] developed a satellite-based approach, which integrated forest inventory data at the provincial level with synchronous normalized difference vegetation index (NDVI), to estimate the spatial distribution of forest biomass from 1982 to 1999 in China. However, this approach that relied on forest inventory data at the provincial level could miss information on the variability of biomass density within forest types. To refine China-wide mapping of aboveground forest biomass, a direct combination of remote sensing and forest inventory data at the plot level is then necessary. This will leverage a combination of forest inventory data that provide accurate information at the plot level, and remote sensing data that are continuous in time and space.

In this paper, we estimate aboveground biomass across China through combining forest inventory plot data over 348 sites [18] with seven spectral reflectance bands from Moderate-Resolution Imaging Spectroradiometer (MODIS) sensors. MODIS spectral reflectance bands are considered because they are more accurate in predicting aboveground biomass than NDVI only having two reflectance bands [19]. Thus, the main objectives of this study are: (1) to quantify the spatial patterns of forest above ground biomass in China, (2) to explore the spatial relationships of forest above ground biomass with climate factors (temperature and precipitation), and (3) to assess change in China’s forest above ground biomass since 2000s.

## Data and Methods

### Forest aboveground biomass density data

**Fig 1:**
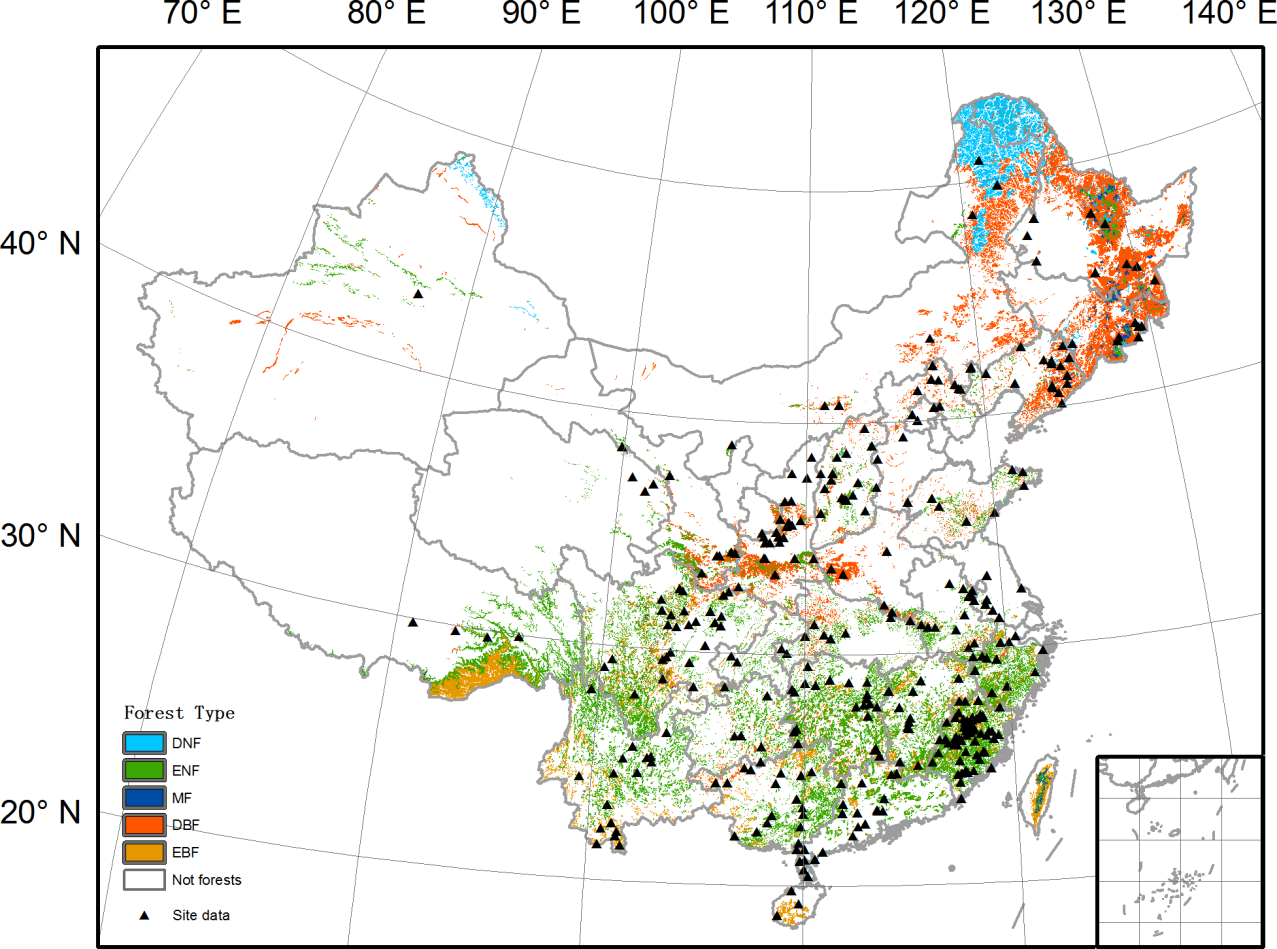
Forest types and the distribution of AGBD data in China. The forest types are according to the 1:4000000 vegetation map of China. DNF = deciduous needle leaf forests, ENF = evergreen needle leaf forests, MF = needle leaf and broadleaf mixed forests, DBF = deciduous broadleaf forests, EBF = evergreen broadleaf forests. The AGBD data is from Luo *et al*. [18].

The forest aboveground biomass density (AGBD) data used in this study is a collection of published ground measurements from 348 sites across China (Fig 1) during 1978–2008 [18]. All these data have been checked using criteria following Ni et al. [19] to ensure their validity. The final dataset provides 1607 AGBD records with tree species, latitude and longitude information. AGBD values from this dataset vary from 1.87 Mg ha^-1^ to 1433.21 Mg ha^-1^ with 80% falling between 26 Mg ha^-1^ and 187 Mg ha^-1^. The average of the AGBD data is 92.35 Mg ha^-1^. The area of sample plots varies from 100–400 m^2^ for boreal and temperate forests to 1000–2000 m^2^ for tropical forests [19]. To match the gridded satellite-based reflectance data at a spatial resolution of 1 km, the ground-measured AGBD data within each 1 km pixel were averaged and used in further analyses. The unit of AGBD data (Mg ha^-1^) was converted to Mg C ha^-1^ by multiplying a factor of 0.5 [10].

### MODIS data

The MODIS nadir bidirectional reflectance distribution function adjusted reflectance (NBAR) product (MCD43B4, obtained from https://lpdaac.usgs.gov/) is used for forest aboveground biomass estimation in this study. This product provides seven-band reflectance at wavelengths from 459 to 2155 nm (Table 1) since 2000, with a spatial resolution of 1 km and a temporal resolution of 8 days. This cloud-screened dataset has been corrected for view geometry and atmospheric attenuation. Due to its ability to capture forest growth conditions, this product has been successfully applied to predict forest biomass in Russia [14] and Africa [20]. We used summer (June to August) mean reflectance as an explanatory variable of forest biomass since the use of winter reflectance data in snow-covered regions may lead to erroneous biomass estimation.

**Table 1:** Explanatory variables used in MTE.

Variable	Time	Variable type
MODIS band 1 (459–479 nm)	2001–2013 summer^1^	Regression and split
MODIS band 2 (841–876 nm)	2001–2013 summer	Regression and split
MODIS band 3 (545–565 nm)	2001–2013 summer	Regression and split
MODIS band 4 (620–670 nm)	2001–2013 summer	Regression and split
MODIS band 5 (1230–1250 nm)	2001–2013 summer	Regression and split
MODIS band 6 (1628–1652 nm)	2001–2013 summer	Regression and split
MODIS band 7 (2105–2155 nm)	2001–2013 summer	Regression and split
NDVI	2001–2013 summer	Regression and split
EVI	2001–2013 summer	Regression and split
Latitude	—	Split
Longitude	—	Split
Forest type	—	Split

^1^ The 2001–2013 summer values of MODIS reflectance and Vegetation Indices are calculated by averaging values from June to August during 2001–2013.

Vegetation indices including normalized difference vegetation index (NDVI) and enhanced vegetation index (EVI) are good proxies of vegetation greenness and have been widely used to estimate forest biomass [17,21,22]. The explanatory variables used for biomass estimation also include 1 km 16 day NDVI and EVI data during the summertime from MOD13A2 product (obtained from https://lpdaac.usgs.gov/) that can complement single-band reflectance values. The vegetation indices are also considered because the relationships between vegetation indices and single band reflectance could be non-linear, although vegetation indices are calculated based on the reflectance data.

### Climate data

Monthly temperature and precipitation data are from WorldClim dataset with a spatial resolution of 1 km [23]. The monthly temperature and precipitation were aggregated into mean annual temperature (MAT) and mean annual precipitation (MAP) in order to investigate responses of MTE-derived AGBD to climatic factors on the spatial scale.

### Forest distribution map

China has a total forest area of 150 Mha, almost including all major forest types in Northern Hemisphere. Due to a strong precipitation gradient from east to west in China, forests are mainly distributed in the eastern part of China (Fig 1). In northwest China, forests are only distributed in middle or upper parts of mountains due to water limitation. Forests types and their spatial distributions used in this study are based on the 1:4000000 vegetation map of China [24]. This dataset has a total of 175 forest types, which are further aggregated into five main types given the fact that ground measurements of AGBD in all forest types are not fully available. These five types are deciduous needle leaf forests (DNF), evergreen needle leaf forests (ENF), needle leaf and broadleaf mixed forests (MF), deciduous broadleaf forests (DBF), and evergreen broadleaf forests (EBF).

### Data analysis

**Fig 2:**
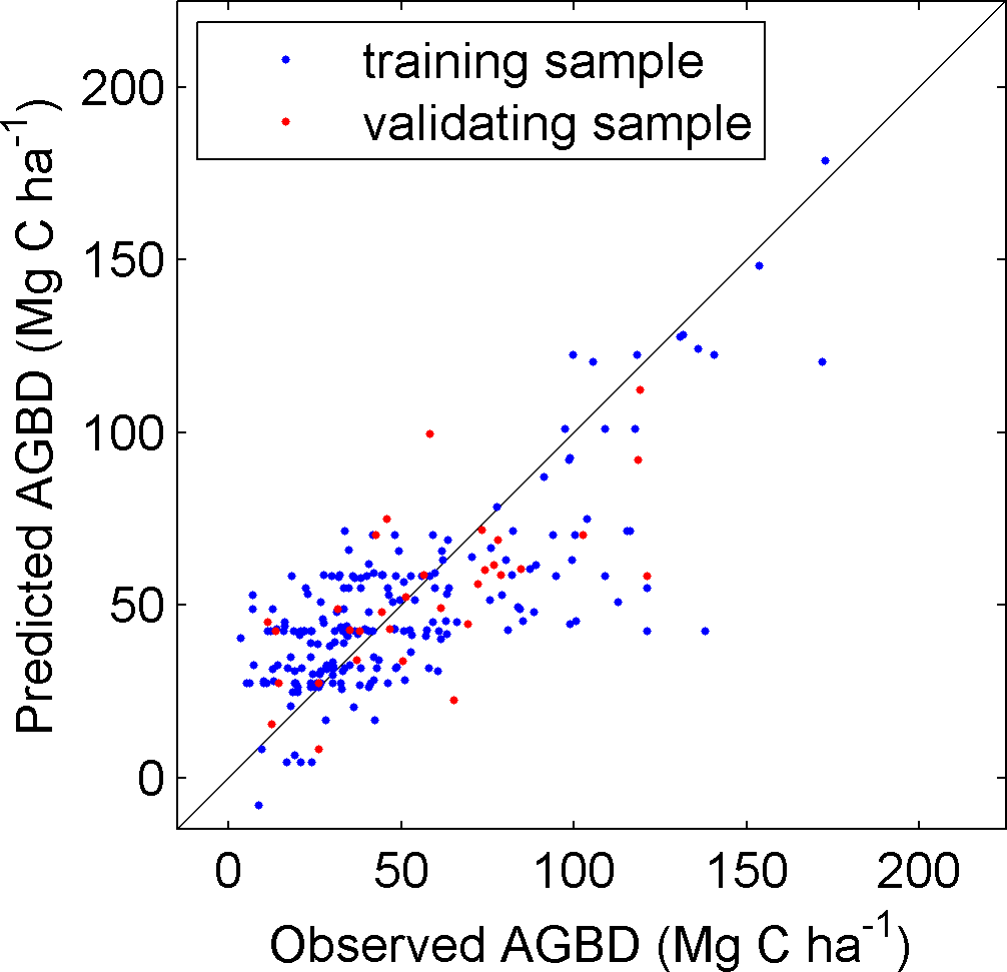
Comparison of observed AGBD (Mg C ha^-1^) against predicted AGBD using MTE algorithm. The blue dots indicate the training samples (R^2^ = 0.57, RMSE = 22.4 Mg C ha^-1^), and the red ones refer to the validation samples (R^2^ = 0.46, RMSE = 22.7 Mg C ha^-1^).

A machine-learning technique Model Tree Ensembles (MTE) [25] is used to predict grid-scale forest aboveground biomass density based on ground-measured AGBD and remote sensing data in China. In this study, the MTE was trained with ground-measured AGBD as the dependent variable and the set of AGBD explanatory variables listed in Table 1 as inputs. 90% of the ground measured AGBD data is used in the training phase and the rest 10% is used for validation. As shown in Fig 2, the AGBD prediction using MTE explained half of the AGBD variation (R^2^ = 0.57, RMSE = 22.4 Mg C ha^-1^ for the training data, and R^2^ = 0.46, RMSE = 22.7 Mg C ha^-1^ for the validation data). We then extended the trained MTE to the whole China. For each forest pixel (1 km in our case), AGBD is estimated from the trained MTE based on satellite-derived reflectance and vegetation indices (Table 1) during the period from 2001 to 2013.

To investigate the relationships between AGBD and climate factors, we classified all the forest pixels into 1°C MAT and 100 mm y^-1^ MAP bins, and calculated the average AGBD of each bin in a MAT-MAP space. To further investigate how forest AGBD varies with precipitation and temperature respectively, linear regressions were performed to calculate the sensitivity (slope) of AGBD to precipitation (SP) in each 1°C temperature bin, and the sensitivity of AGBD to temperature (ST) in each 100 mm y^-1^ precipitation bin.

In addition, we also calculated the difference of forest AGBD between the periods of 2011–2013 and 2001–2003 to represent the total aboveground biomass C stock change of Chinese forests over the past decade. We did not consider forest area change because of a lack of high resolution deforestation and afforestation data.

## Results

### Total forest aboveground biomass C stock and its spatial distribution

During the period 2001–2013, the total AGB of Chinese forests is estimated to be 8.56 Pg C, with an average AGBD of 56 Mg C ha^-1^ over a forest area of 153 Mha (Table 2). This total forest C stock is mainly contributed by ENF and DBF, which account for 41.5% (3.55 Pg C) and 30.4% (2.60 Pg C) of total forest AGB respectively. By contrast, other three forest types (EBF, DNF and MF) have a lower AGB and only account for 17.4%, 8.4% and 2.5% of the country’s total forest AGB (Table 2). The high AGB of ENF and DBF can be mainly attributed to their large area of 68.7 Mha and 48.7 Mha, which occupies 45% and 31.9% of total forest area in China respectively. In contrast, AGBD is not likely to contribute to the high AGB in ENF and DBF since their AGBD (51 and 53 Mg C ha^-1^) are lower than the average AGBD of all Chinese forests (56 Mg C ha^-1^).

**Table 2:** Area and aboveground biomass characteristics for five forest types in China during 2001–2013.

Forest type	Area (Mha)	Total AGB (Pg C)	Average AGBD (Mg C ha^-1^)	Median AGBD (Mg C ha^-1^)
DNF	12.7 (8.30%)	0.72 (8.40%)	56.6	56.9
ENF	68.7 (45.00%)	3.55 (41.50%)	51.6	42.7
MF	2.2 (1.40%)	0.21 (2.50%)	97.4	63.0
DBF	48.7 (31.90%)	2.6 (30.40%)	53.3	42.7
EBF	20.4 (13.40%)	1.49 (17.40%)	73	48.4
All forests	152.6 (100%)	8.56 (100%)	56.1	42.7

DNF = deciduous needle leaf forests, ENF = evergreen needle leaf forests, MF = needle leaf and broadleaf mixed forests, DBF = deciduous broadleaf forests, EBF = evergreen broadleaf forests.

**Fig 3:**
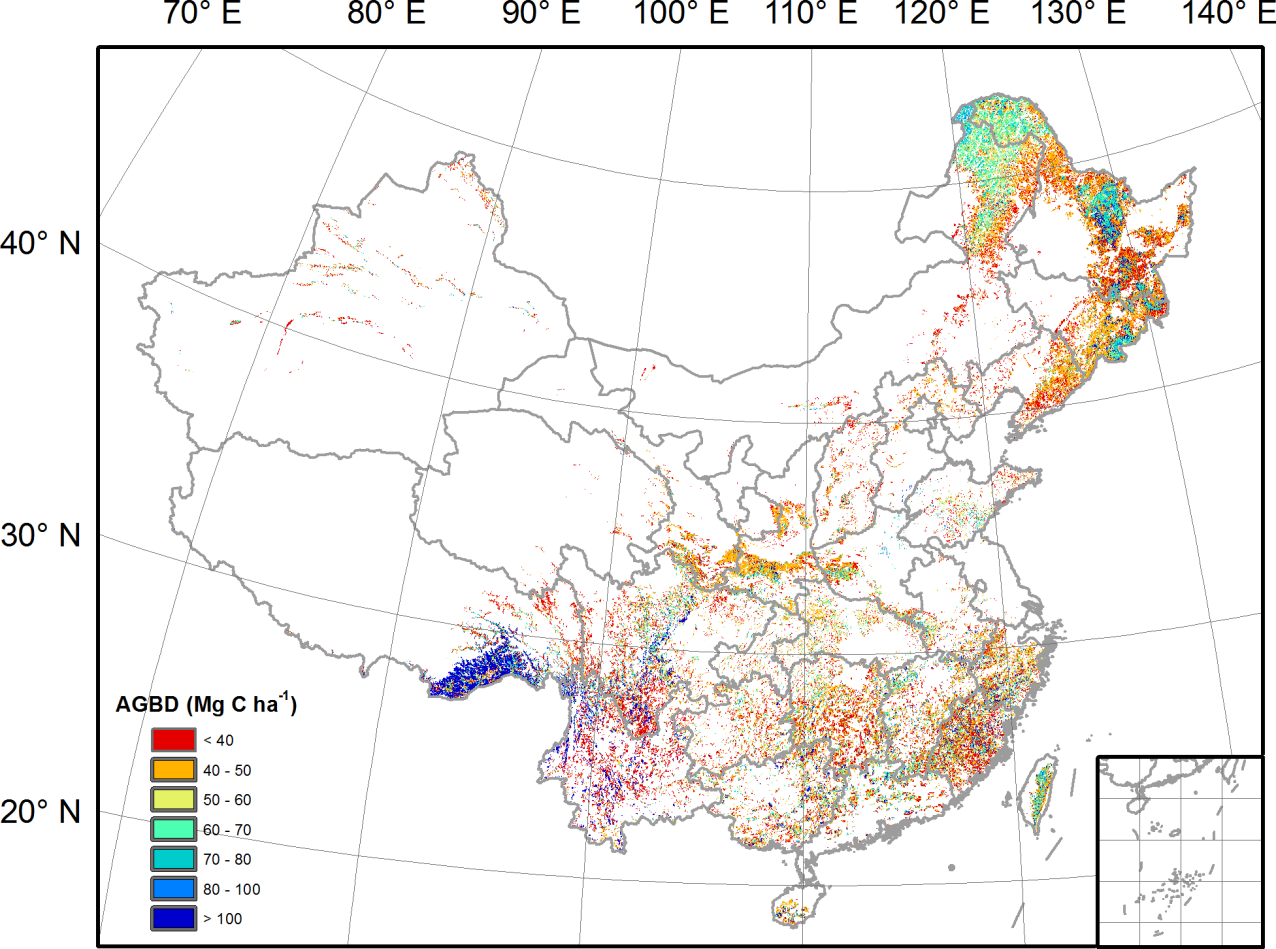
Spatial distribution of mean forest AGBD during 2001–2013.

Fig 3 illustrates the spatial distribution of forest AGBD across China, indicating a strong spatial heterogeneity. More than half of Chinese forest AGBD falls in a range of 40–70 Mg C ha^-1^. The highest AGBD was found in tropical rainforests in southeastern Tibet with values higher than 100 Mg C ha^-1^. Besides southeastern Tibet, central areas of Mts. Daxinganling, Mts. Xiaoxinganling and Mts. Changbai in Northeast China also show a relatively high AGBD, with values ranging from 60 to 80 Mg C ha^-1^. However, the edge regions of these mountains display a relatively low AGBD (< 40 Mg C ha^-1^). The relatively low AGBD can also be found in South China, Xinjiang and central Inner Mongolia.

### Spatial relationships between forest AGBD and climatic factors

**Fig 4:**
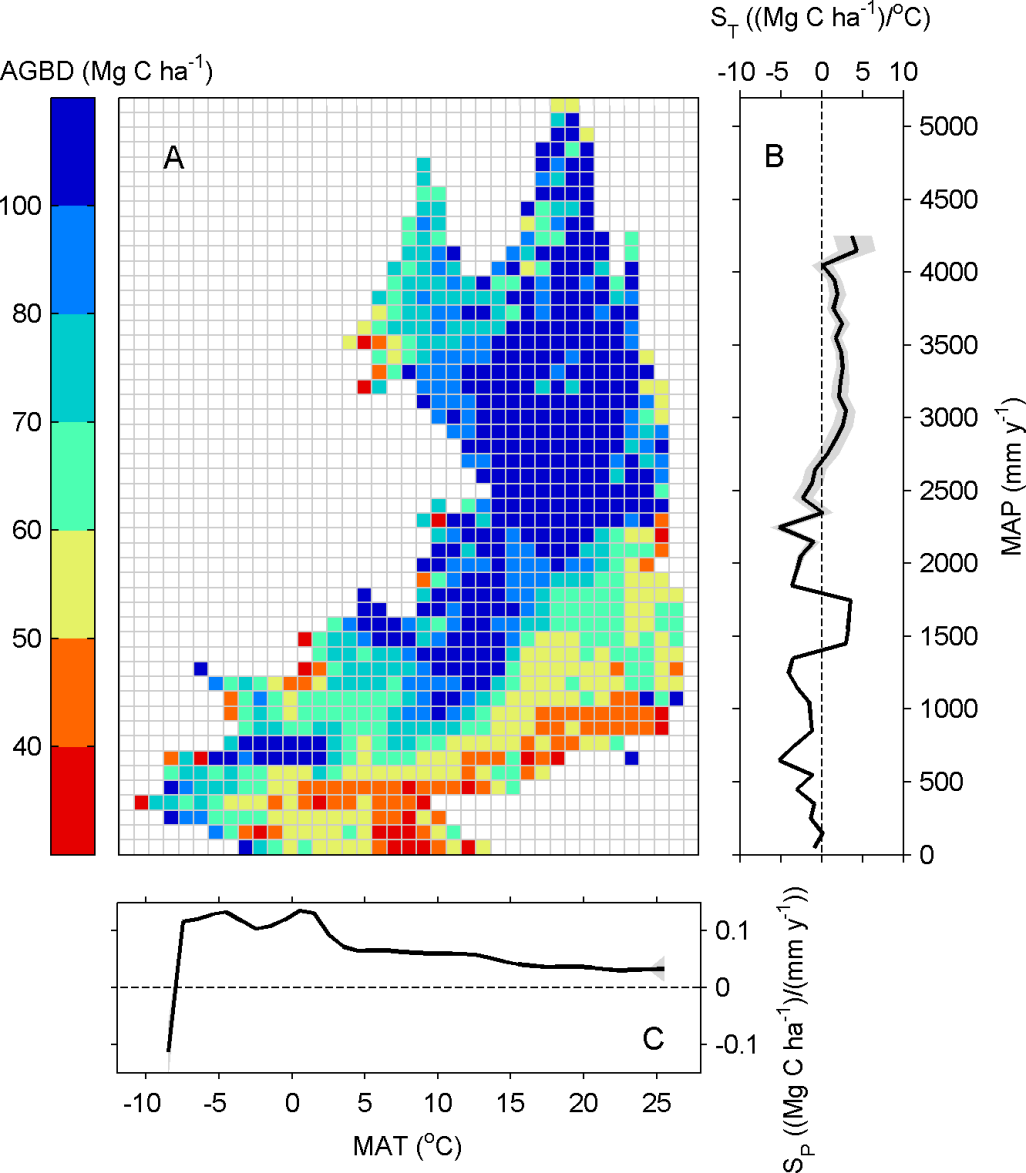
(A) Distribution of forest AGBD in a two-dimensional space with (MAT) and (MAP) binned into intervals of 1°C MAT and 100 mm MAP. **(B) The sensitivity of AGBD on temperature (S**_**T**_ **) along precipitation gradient. (C) The sensitivity of AGBD on precipitation (S**_**p**_ **) along temperature gradient.** The shaded area in (B) and (C) indicates 95% significance intervals of S_T_ and Sp. Sensitivities were only calculated in bins having more than 100 grid pixels.

As shown in Fig 4, forests in China are distributed in regions with MAT ranging from -10 to 26°C and MAP ranging from 0 to 5200 mm y^-1^. The highest AGBD levels (> 100 Mg ha^-2^) are mostly found in temperate (5–22°C) and moist climate regions (MAP > 1000 mm y^-1^), while the relatively low AGBD levels (< 40 Mg ha^-2^) mainly occur in regions with MAP < 500 mm y^-1^ and MAT > 5°C.

To further investigate the relationships between AGBD and climatic factors, S_P_ along the MAT gradient and S_T_ along the MAP gradient were explored. Along the MAT gradient, S_P_ is always positive except in regions with MAT less than -8°C. As MAT increases from -8 to 25°C, the value of S_P_ gradually decreases from 0.1 to 0.03 (Mg C ha^-1^)/(mm y^-1^). By contrast, S_T_ tends to increase with MAP. For example, S_T_ shows negative values from 0 –-5 (Mg C ha^-2^ °C^-1^) in regions with MAP less than 1300 mm y^-1^ and displays positive values of 0–5 (Mg C ha^-2^ °C^-1^) in regions with MAP higher than 2800 mm y^-1^.

### Change in forest aboveground biomass C stock

**Fig 5:**
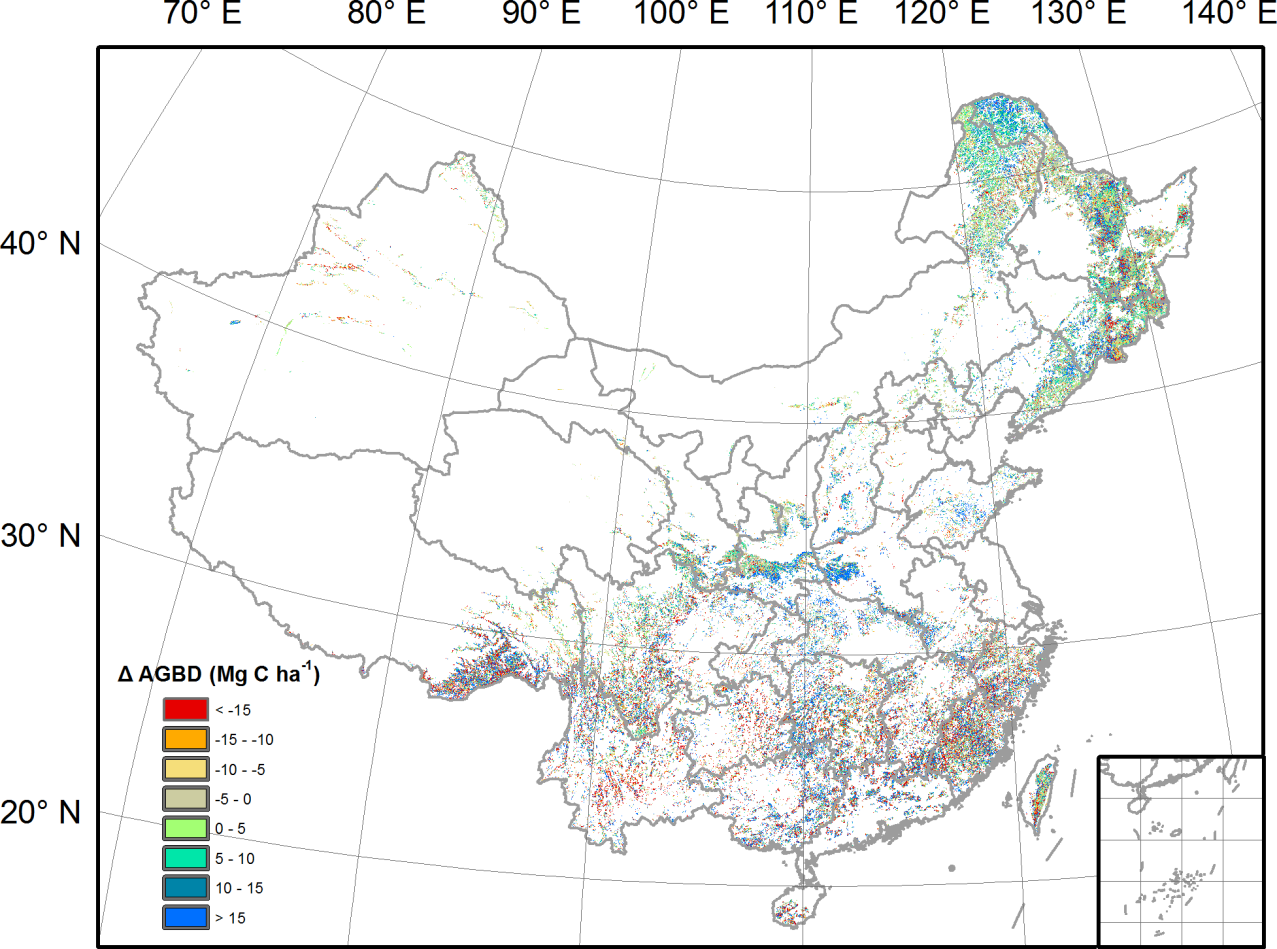
Spatial distribution of the change in forest AGBD. The change in AGBD is calculated as the difference between the period 2011–2013 and the period 2001–2003.

Over the past decade, mean Chinese forest AGBD has increased by 4.6 Mg C ha^-1^, resulting in an increase of aboveground biomass C stock by 61.9 Tg C y^-1^. However, changes the in forest AGBD display large spatial heterogeneity (Fig 5). In regions north of 30°N, about 65% of forests show an increase of AGBD. The highest increase of AGBD is located in mountains of the northeast China and Mts Qinling, with values larger than 10 Mg C ha^-1^. The total aboveground biomass C sink in this region is 47.4 Tg C y^-1^, accounting for 77% of Chinese total forest aboveground biomass C sink. By contrast, forests located south of 30°N showed a mosaic pattern of the increase and decrease in AGBD. A widespread increase of AGBD is mainly found in Hunan and Guangxi provinces, and a large-area decrease is found in Yunnan and Guizhou provinces. The total aboveground biomass C sink in this region is 14.5 Tg C y^-1^, occupying 23% of the whole country’s forest biomass C sink.

## Discussion

Over the past decade, many estimations of forest biomass have been made based on satellite reflectance observations [13,20,26,27]. By integrating MODIS reflectance and ground-measured AGBD in a machine-learning algorithm (the model tree ensemble; MTE), we estimated that China’s total forest AGB was about 8.56 Pg C during 2001–2013. This value is lower than that in the USA (42.3 Pg C) based on MODIS land product combined with foliage-based generalized allometric models [12] (forests area from [28]), and that in Canada (17.8 Pg C) using the combination of inventory photo plots, MODIS spectral data and climate data [29]. Much higher total AGB in the USA is mostly contributed by their larger AGBD. For example, at the country scale, In terms of mean forest, our estimation of AGBD in China is about 56 Mg C ha^-1^, which is only 40% of that in the US (141.1 Mg C ha^-1^; [12]), but is comparable to that in Canada (62.02 Mg C ha^-1^; [29]). While many studies have investigated total forest biomass in China, few have estimated total AGB. Based on provincial wood-volume inventory data and conversion factors, previous studies have estimated that the total forest biomass of China is 3.76–6.2 Pg C [30]. However, our estimate of AGB is larger (8.56 Pg C). This large difference between our forest biomass estimate and those of previous studies may be because we used a new dataset of AGBD measurements rather than previously used provincial forest inventory data. Regardless, these discrepancies highlight the need to reduce uncertainties in biomass measurements.

Spatially explicit assessments of forest biomass are critical to the design and implementation of effective sustainable forest management strategies and forest policies [15,16]. Taking advantage of the image-based spatial information from remote-sensing, previous studies have mapped high resolution AGBD in different regions [12,16,29]. In this study, we found the highest AGBD in regions with moderate high precipitation and cool temperature (e.g. southeastern Tibet and northeastern China), and relatively low AGBD in dry inland regions. Similar spatial pattern of AGBD with higher values located in humid regions is also observed in the USA, Canada and Europe [12,16,29]. Moreover, we also found that AGBD increase with MAP in almost all MAT ranges (Fig 4), which is consistent with the global site analysis by Keith et al. [31] and supports the finding that adequate rainfall favors the rapid growth of forest [31,32]. Regarding the impact of temperature on AGBD, Larjavaara and Muller-Landau [33] reported that temperature explains most of variation in aboveground biomass among humid old-growth forests. Stegen et al. [32] found very weak AGBD responses to MAT in tropical and temperate forests. In this study, the responses of AGBD to MAT are closely bound up with water conditions. Significant negative temperature sensitivities of AGBD dominate the regions with MAP less than 1300 mm y^-1^, which can be attributed to higher transpiration rates and stomatal closure induced by higher vapor pressure deficit. By contrast, positive temperature sensitivities of AGBD are observed in wet regions with MAP larger than 2800 mm y^-1^ where forest growth is scarcely constrained by water but by energy.

In addition to the impact of climate on spatial distribution of AGBD, anthropogenic activities also play a crucial role in determining the magnitude of AGBD. We noted that relative low AGBD located in southwest and southeast regions (Fig 2) cannot be explained by their climate conditions since which are much more favorable for forest growth. Instead, such relatively low AGBD may be closely related to young stand age. Owing to national afforestation campaigns since the end of 1970s, a large area of forests in south and southeastern China has a stand age less than 40 years [34]. Although changes in total forest biomass can be affected by land use change [35,36], NPP that is critical to forest biomass C accumulation is mainly affected by climate instead of land use change in temperate regions [37].

In this study, we found an overall increase rate of 61.9 Tg C y^-1^ in Chinese forest aboveground biomass C during 2001–2013 According to a recent estimation of Chinese total forest biomass C sink of 115 Tg C y^-1^ over the past decade [35], our result suggests that forest aboveground biomass can contribute about 53.8% of total forest C sink. The cumulative aboveground biomass C sink from 2001 to 2013 (0.80 Pg C) can offset 4.7% of cumulative anthropogenic C emissions in China during the period 2001–2010 (17.0 Pg C) [38]. According to the spatial pattern of changes in AGBD, aboveground forest biomass C sink is mainly located in northeast China and Mts. Qinling. This pattern is roughly consistent with the spatial distribution of total forest C sink during the period 2001–2010 estimated by Peng [30]. Different factors are responsible for the changes of forest AGBD in different regions. For northeastern and central China, forest C accumulation could benefit from warming climate that is accompanied with a longer growing season [39], increased photosynthesis rate, and stimulated nitrogen mineralization. But this warming-induced C sink becomes smaller and even vanishes in western China with low precipitation since high temperature can impose serious water stress on forest growth. Moreover, increasing atmospheric CO_2_ also favors forest aboveground biomass C sink due to the stimulation of CO_2_ fertilization effect [40,41]. In addition, China has experienced accelerated nitrogen (N) deposition because of rapid industrial and agricultural development, especially in 2000s [42]. This increased N input is also likely to increase forest C sink particularly in N-limited Asia temperature and subtropical forest ecosystems, which can be inferred from the observed positive spatial correlation between NEP and N deposition across Asian forest sites [43]. Furthermore, model simulations by Lu et al. [44] have also indicated that accelerated N deposition in China leads to the increased forest C storage during the period of 2001–2005. However, in a majority of the southern regions, forest AGBD significantly reduced during the period of 2000–2013. For the southwestern regions, drought-induced high tree mortality rate is mainly responsible for the aboveground forest biomass C sources [45,46].

There are considerable uncertainties in our estimates as the forest map used in this study was digitalized in 1996 [24]. Because no detailed information of deforestation and afforestation is available, changes in the forest area since then are not considered, and this may lead to underestimates of AGB and C sink due to the increasing forest area in China [28]. However, uncertainties are expected to decrease as new forest maps and more accurate deforestation and afforestation data becomes available.

Uncertainty also came from AGBD measurements. In most plots, AGBD was determined by either using the biomass of several standard trees for other trees, or by applying the standard trees’ biomass-diameter at breast height-tree height relationships to other trees [19]. These techniques may have contributed to errors in AGBD, especially in plots with highly variable tree sizes. Furthermore, the plot size of AGBD measurements varied from 100 m^2^ to 2000 m^2^, while the pixel of remote sensing data was 1 × 1 km. This difference in area produced considerable uncertainty due to the large heterogeneity of the forest at a small scale [47]. To reduce uncertainty, a well-designed network to measure forest biomass in larger plots is needed.

Remote-sensing data also introduced uncertainty into our estimation. We used MODIS reflectance and vegetation indices to estimate AGBD. Although these data are well calibrated, small variations caused by atmospheric effects and illumination geometry may exist in these datasets. In addition, the reflectance and vegetation indices are unable to fully capture forest structure information under a closed canopy. Fortunately, new technology is expected to reduce this uncertainty, such as light detection and ranging (LiDAR), which is an active remote-sensing technique based on laser light that improves estimates of canopy vertical structure. This information is then translated to a more-accurate aboveground biomass estimate [48–50]. Moreover, the 1 × 1-km MODIS data were available only after 2000, while AGBD measurements were obtained between 1978 and 2008 [18]. Therefore, we must assume that, when compared with the spatial variation of AGBD, changes in MODIS data over time are negligible. This uncertainty is expected to decrease as more AGBD data are measured in the future.

## Conclusions

Combining data from field sampling and the satellite image variables we produced high resolution maps of above ground forest biomass in China. We estimated that during 2001–2013, total AGB of forests in China was 8.56 Pg C and that the mean AGBD was 56.1 Mg C ha^-1^. The distribution of AGBD was closely related to MAP and MAT, and generally AGBD increased with MAP along all temperature gradients. In addition, AGBD increased with MAT in regions with MAP higher than 2800 mm y^-1^ and decreased with MAT in regions with MAP less than 1300 mm y^-1^. By investigating differences in AGBD from 2001–2003 and 2011–2013, we estimated that during the last decade, forests in China sequestered C in aboveground biomass at a rate of 61.9 Tg C y^-1^. This C sink is distributed mainly in northern China and may be attributed to a warming climate, rising CO_2_ concentration, N deposition and growth of young forests.

Compared with previous studies, our estimates, which combined data from field sampling and satellite images, provide another means of evaluating the aboveground biomass of forests. Although uncertainties exist within our estimates, they are expected to decrease as further field data become available, new remote-sensing technology is developed, and forest distribution maps are updated. In addition, gaining a deep understanding of total forest biomass stock and its dynamics also requires improved knowledge of below ground biomass that stores a large part of total carbon stocks. However, the quantification of below ground biomass is challenging because it cannot be detected by satellite observations. This necessitates an increased density of in situ measurements and improved scaling algorithms in the future study.
